# Synergistic Effects of Polydopamine/Medical Stone Bio-Adsorbents for Enhanced Interfacial Adsorption and Dynamic Filtration of Bacteria

**DOI:** 10.3390/polym16213027

**Published:** 2024-10-29

**Authors:** Wenfeng Chen, Sha Wan, Hongxin Lin, Shimi Li, Anhua Deng, Lihui Feng, Yangfan Xu, Xu Zhang, Zhen Hu, Fang Xu, Kun Yan

**Affiliations:** 1 CCCC Second Harbor Engineering Company Ltd., Wuhan 430040, Chinazhanglaohan4601@foxmail.com (X.Z.); 2 Key Laboratory of Textile Fiber & Product, Ministry of Education, Wuhan Textile University, Wuhan 430200, China; 3 Wuhan Huzhenyu Environmental Technology Company Ltd., Wuhan 430000, China; ahuzhen@126.com; 4 Wenzhou Haichen Technology Development Company Ltd., Wenzhou 325700, China; 18958979696@163.com

**Keywords:** medical stone, polydopamine nanocoating, adsorption, filtration, disinfection

## Abstract

Polymer-based wastewater disinfection, which is typically performed using chemical oxidation or irradiation, can result in various toxic byproducts and corrosion under harsh environments. This study introduces a robust bio-adsorbent prepared from naturally abundant polydopamine-modified medical stone (MS@PDA) for the high-efficiency removal of bacteria from water. The PDA nanocoating can be easily applied through an in situ self-polymerization process, resulting in a considerably high bacterial adsorption capacity of 6.6 k pcs mm^−2^ for *Staphylococcus aureus*. A cyclic flow-through dynamic filtration and a disinfection system was implemented using an MS@PDA porous filter with an average pore size of 21.8 ± 1.4 µm and porosity of ~83%, achieving a 5.2–6.0-fold enhancement in the cumulative removal efficiency for MS@PDA_2_. The underlying mechanisms were elucidated through the synergistic effects of interfacial bio-adsorption and size-dependent interception. Notably, the bacteria captured on the surface could be killed using the enhanced photothermal effects of the PDA nanocoating and the inherent antimicrobial properties of the mineral stone. Thus, this study not only provides a new type of advanced bio-adsorbent but also provides new perspectives on an efficient and cost-effective approach for sustainable wastewater treatment.

## 1. Introduction

In recent decades, waterborne microorganisms in wastewater have received increasing attention as a public health concern and have caused numerous deaths globally owing to their environmental damage, morbidity, and mortality [1,2]. Currently, various physical and chemical methods have been developed for removing bacterial pathogens and disinfecting wastewater, including the extensive use of antibacterial agents to kill bacteria and/or viruses present in wastewater [3,4,5]. However, the extensive use of antibacterial agents entails high costs and can easily result in secondary pollution (e.g., metals) and drug resistance (e.g., antibiotics). Therefore, a pressing need exists for the development of a highly efficient universal method for the removal of bacterial pathogens in wastewater treatment and environmental protection [6,7,8]. Recently, advancements in material sciences and technologies have led to the creation of high-performance bio-adsorbents that capture and eliminate bacteria from wastewater via dynamic physical and chemical interfacial interactions [9,10]. For instance, polymer films, COFs/MOFs, porous scaffolds, and filter paper have often been used to absorb and/or separate bacteria from contaminated water [11,12,13]. Most of these materials demonstrate high removal efficiencies; however, the stringent demands for low-cost, environmentally friendly, and scalable production of such materials have restricted their practical applications. Consequently, considerable interest exists in developing green and sustainable bio-adsorbents tailored for the environmental sector.

Medical stone (MS), known to be a typical abundant natural resource, is a type of compound or medicinal mineral rock with considerable potential applications across various fields, including medicine, household tools, decoration, and environmental protection [14,15]. MS usually comprises granite, syenite granite, and quartz, which are found in weathered and partially weathered rock products. Its detailed composition is as follows: SiO_2_ 67.2%, Al_2_O_3_ 16.16%, Fe_2_O_3_ 2.72%, CaO 2.42%, MgO 1.44%, TiO_2_ 0.056%, Ba 0.228%, MnO 0.052%, P_2_O_5_ 0.145%, Na_2_O 5.34%, K_2_O 2.99%, FeO 0.47%, and Br 0.148% [16]. Compared with many current, efficient adsorbents, such as zeolites, MS has several advantages for use as a mineral adsorbent, such as abundant availability, low cost, good stability, and safety for humans, owing to its nontoxic nature [17]. Moreover, MSs often possess substantial specific surface areas, high cation-exchange capacity, and unique permeable properties [18]. Recently, extensive research has been performed on MS across various fields, such as mineralization, antibacterial application, pollutant adsorption, wastewater treatment, and on its role as a biofilter to reduce gaseous emissions and nitrogen losses [19]. However, previous studies have primarily focused on enhancing the antibacterial properties of MS by integrating various organic and/or inorganic antibacterial agents, with limited attention given to its surface adhesion properties for bacteria. This oversight may be related to its mineral characteristics and inherent antimicrobial properties. Therefore, it is desirable to develop a facile surface modification approach to construct multifunctional MS materials (e.g., MS materials with enhanced surface adhesion and antibacterial properties) for a sustainable wastewater treatment system.

Polydopamine (PDA) coatings, inspired by biological adhesive mechanisms, offer remarkable versatility owing to their capability for substrate-independent functionalization with a diverse array of amine- and thiol-containing molecules [20,21]. Numerous studies have demonstrated that PDA-based materials can be used to prepare durable superhydrophobic structures and adhere to a wide variety of surfaces, including glass, fabrics, and metal substrates [22,23,24]. Furthermore, the presence of abundant active groups (e.g., hydroxyl and amino) in PDA provides several advantages, such as increasing the number of binding sites for the covalent attachment of target molecules and facilitating the formation of multifunctional nanocomposites through Schiff base reactions and strong chelation interactions with metal ions [25,26]. Consideration of these factors inspires the innovative development of a high-performance MS-based bio-adsorbents for effective and long-term wastewater treatment. This can be achieved by employing PDA as an intermediate coating to immobilize bacteria on MS substrates, which is followed by in situ killing of bacteria through the inherent antimicrobial properties of PDA.

Herein, a bio-adsorbent and porous filter device based on a naturally abundant MS has been developed for high-efficiency treatment of bacteria-infected water. In particular, MS was selected as a solid substrate to evaluate bacterial adsorption performance. PDA was incorporated to enhance the bio-adsorption capabilities for bacteria (e.g., *Escherichia coli* (*E. coli*), and *Staphylococcus aureus* (*S. aureus*)) via self-polymerization on the MS surface, resulting in PDA-active nanocoating with excellent adhesion properties. Furthermore, a cycling filtration experiment was performed using a PDA-modified MS-based filter to validate the considerable potential of the MS@PDA-based porous filter device for the continuous removal of bacteria from wastewater. The synergistic mechanisms that contribute to the high-efficiency treatment of bacteria-contaminated wastewater through interfacial bio-adsorption and continuous filtration were clarified. Thus, this study may inspire new developments in the creation of advanced bio-adsorbents and filtration devices for the removal of bacteria from wastewater.

## 2. Materials and Methods

### 2.1. Materials

MSs with a mesh size of 50–100 mesh used in this study were purchased from Chuzhou Jingjin Crafts Co., Ltd., Anhui, China. The stones were milled to achieve various sizes using different meshes. *E. coli* (O157:H7) and *S. aureus* (ATCC 29213) were purchased from Nanjing Lezhen Biotechnology Co., Ltd., Nanjing, China. The dopamine (DA) monomers, glutaraldehyde, acridine orange, ethidium bromide, and alcohol were purchased from Sinopharm Chemical Reagent Co., Ltd, Shanghai, China. All chemicals were used as received without any further purification.

### 2.2. Preparation of PDA-Modified Medical Stones

PDA-modified MSs were prepared via an in situ adsorption and self-polymerization method [22]. The detailed process is as follows. First, all MS were thoroughly washed three times with distilled water and then collected using a centrifuge method. The PDA modifications were performed by immersing 15 g of MS in 100 mL of a Tris–HCl buffer solution (10 mM, pH = 8.5), which contained 15 mL of dopamine monomer solution at a concentration of 1 mg/L. The mixture was magnetically stirred at room temperature for approximately 5 h. The MSs soaked in the PDA solution for durations of 0.5, 1, 2, 3, and 4 h were labeled as MS@PDA_0.5_, MS@PDA_1_, MS@PDA_2_, MS@PDA_3_, and MS@PDA_4_, respectively. The untreated MS served as the bare control. After the modification, the samples were purified through centrifugation, and the collected precipitates were gently rinsed three times with distilled water before being vacuum-dried at 80 °C for 2 h for further measurements.

### 2.3. Investigation of the Bio-Adsorption Capacity

The bio-adsorption performances were assessed using two model bacteria: *Staphylococcus aureus* (*S. aureus*) and *Escherichia coli* (*E. coli*). Briefly, the bacteria were transferred and incubated in an LB medium at 37 °C overnight. For visual characterization, all bacterial strains were stained using an acridine orange/ethidium bromide (AO/EB) fluorescent nuclear staining method, as outlined in previous reports [27]. Briefly, 10 mL of bacterial suspension with an optical density (O.D.) value of 1.0 was mixed with the prepared staining solution for 15 min, followed by collection and rinsing with buffer solution three times. For the adsorption measurement, 200 µL of *Staphylococcus aureus* and *Escherichia coli* at a concentration of 10^8^ CFU/mL were added to a 5 mL buffer solution, and the mixture was magnetically stirred with 0.25 g of MS@PDA at 100 rpm for 10 min. The O.D. of the resultant solution at 600 nm was measured at specific time points using UV–vis spectroscopy to determine the bacterial concentration and removal efficiency. An O.D. value of 1 corresponds to 1 × 10^9^ CFU/mL.

### 2.4. Filtration Performance and Removal Efficiency

To evaluate the filtration performance, 1 g of bare MS and MS@PDA samples prepared with varying reaction times were collected and spread on a commercial filter paper substrate. To create a stable and compact stone-based porous membrane, 50 mL of phosphate-buffer solution (0.1 M, pH ~7.0) was used to rinse the particles using a suction method, repeating this process three times. The filtration experiments used a 20 mL solution of *Staphylococcus aureus* at a concentration of 10^8^ CFU/mL and followed a vacuum filtration method at 0.1 Pa. The solutions were filtered for five rounds under consistent conditions, and the O.D. of the resultant solution at each cycle was measured using UV–vis to calculate the overall removal efficiency.

### 2.5. Characterization

Optical images of the MS were captured using a digital camera under natural light conditions. Fluorescence images were obtained with a fluorescence microscope focused on the green channel. The molecular interactions of the nanocomposites were characterized through Fourier transform infrared (FT–IR, Vertex 70, Bruker, Bruker Corporation, Germany) spectroscopy, covering the range of 500–4000 cm^−1^. The crystalline structure was analyzed via X-ray diffraction (XRD, PANalytical X’Pert Pro, PANalytical, Kassel, Germany) using Cu Kα radiation at 40 kV, 40 mA, and a room temperature of 25 °C. The surface morphologies of the MS, both before and after modification, were assessed using scanning electron microscopy (SEM, JEOL JSM-5700F, JEOL Ltd., Tokyo, Japan). All samples that adhered to the bacteria were immobilized in a 2.5 *v*/*v*% glutaraldehyde solution for 5 h, then desiccated in alcohol for 10 min, soaked in liquid nitrogen, and subsequently freeze-dried at −40 °C for 12 h before SEM measurements. The zeta potential was measured using a Zetasizer instrument (Zetasizer Nano ZS, Malvern, UK). The photothermal properties were evaluated by irradiating the nanocomposites with an 808 nm near-infrared light at an output power of 1.0 W. Temperature readings were taken over a 5 min period using a digital thermometer (Fluke Ti400, Shanghai, China). The pore size distributions of the stone-based porous filter membranes were examined via a capillary flow porometer (Model CFP-1500A, PMI Inc., California, USA). The O.D. values were collected via a UV-2700 spectrophotometer (Shimadzu Instruments Co., Ltd., Kyoto, Japan) at a wavelength of 600 nm.

## 3. Results and Discussion

### 3.1. Preparation of MS@PDA for Enhanced Bacterial Bio-Adsorption and Cycling Infiltration

**Figure 1:**
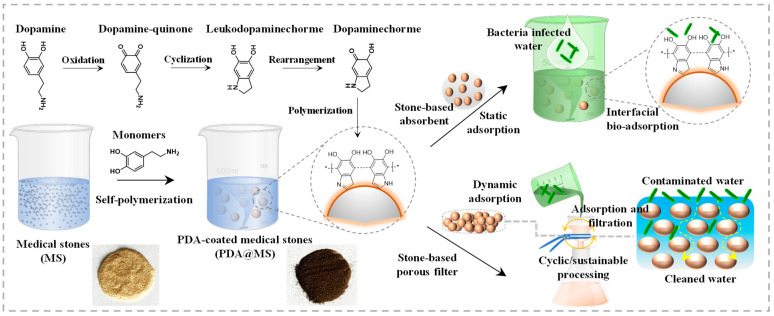
Schematic diagram depicting the preparation of polydopamine-modified medical stones (MSs) designed to enhance interfacial bio-adsorption performance and improve dynamic filtration adsorption efficiency for treating bacterial-infected wastewater (the “*” represents the repeating structural units of the polymer molecule, with the following parts being identical).

The abundant natural resources of MS have frequently been modified to meet the complex requirements of environmental protection. To enhance the interfacial activity of MS, a PDA nanocoating was applied to the MS surface based on the adhesive behaviors of marine mussels. This process relies on the strong interactions between the catechol groups in DA and MS, which occur through chelation, hydrogen bonding, and covalent interactions [26]. The primary concept and potential applications of MS@PDA are schematically illustrated in Figure 1. The nanocoating can be easily applied by immersing MS in a DA solution for a specified duration (e.g., 0–4 h), allowing for the in situ adsorption of DA monomers onto the MS surface followed by self-polymerization under alkaline conditions. After modification, MS was purified via centrifugation and then redispersed in deionized water for further applications. The abundant catechol groups in the PDA nanocoating were expected to endow MS with excellent bio-adsorption properties, making it a versatile bio-adsorbent for removing bacteria from wastewater. However, the static adsorption process of bio-adsorbents is typically performed in a closed system, and the removal efficiency is often constrained by the available amount of the adsorbent and the relatively long contact time required. Given the substantial volume of water treatment in practical applications, a porous MS-based filter was developed to enable dynamic filtration adsorption. In this system, contaminated water was pumped through a porous filter device tightly packed with MS@PDA, allowing for continuous processing. Consequently, this study demonstrates that the bacteria removal efficiency of MS can be enhanced through a PDA nanocoating and by integrating a bacteria-enriched filtration process. The continuous operation was performed using a cyclic dead-end filtration test in conjunction with an interfacial bio-adsorption process that used the tightly packed MS@PDA as a porous filter medium.

### 3.2. Microstructure and Characterization of MS@PDA

**Figure 2:**
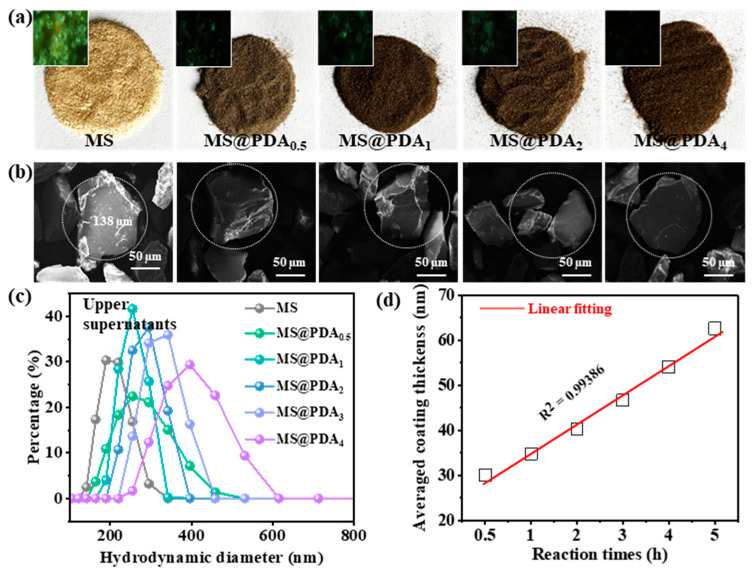
Characterization of MS@PDA samples prepared with varying reaction times (0–4 h). (**a**) Optical images captured at different magnifications. (**b**) Scanning electron microscopy (SEM) analysis. (**c**) Dynamic light scattering (DLS) results of the particle-containing supernatants. (**d**) The thickness of the PDA nanocoating on the MS surface as a function of reaction time; the thickness was calculated from half of the change in the hydrodynamic diameter at the peaks.

The optical images of MS@PDA prepared with various reaction times are shown in Figure 2a. The natural yellow appearance of MS changed dramatically to brown in half an hour, becoming progressively darker over 4 h. This change can be attributed to the oxidation of catechol groups and the formation of PDA nanocoatings from dopamine monomers under alkaline conditions [20]. These findings serve as preliminary evidence for the successful formation of PDA nanocoatings on MS surfaces and indicate that the coating structure (e.g., thickness) is considerably influenced by reaction times. To further investigate the alkali-induced dopamine self-polymerization, the morphologies of the PDA coating were examined using SEM. Figure 2b shows that all powders exhibit an irregular shape with a relatively uniform diameter ranging from 10 to 200 μm. The bare MS features a relatively rough surface, which gradually becomes smooth after modification with the PDA nanocoating. Given that the microsize of MS (~138 µm) exceeds the dynamic light scattering (DLS) measurement range (<6 µm), the variations in nanocoating thickness were assessed from the particle-containing supernatants, with the hydrodynamic diameters of the small particles shown in Figure 2c. The bare sample presents an average size of 180 nm, whereas the particle-containing supernatants demonstrate larger sizes, with hydrodynamic diameters ranging from 200 to 600 nm. These findings can be attributed to the continuous growth of the PDA nanocoating on the MS surface. Figure 2d shows the linear relationship between reaction time and the average thickness of the PDA nanocoating. This section indicates that the PDA nanocoating can be easily prepared on the MS surface using a self-polymerization method, allowing for controlled thickness and a uniform surface microstructure.

**Figure 3:**
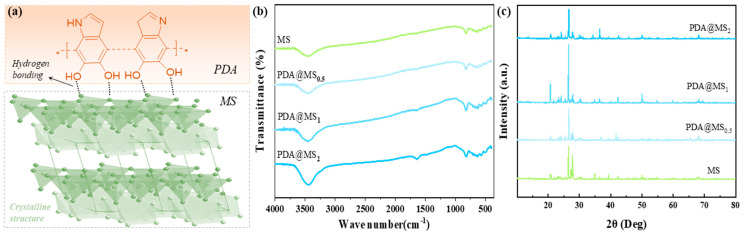
(**a**) Schematic illustration of the hydrogen bonding and crystalline structure of the PDA-modified MS nanohybrids. (**b**) FTIR and (**c**) XRD. “*” represents the repeating structural units of the polymer molecule.

Compared to the polymer-based absorbents, the naturally abundant MS possesses a unique laminated crystalline structure and is expected to exhibit considerable corrosion resistance under harsh conditions. As illustrated in Figure 3a, a strong interfacial interaction (e.g., hydrogen bonding) is expected to develop between MS and PDA owing to its plentiful active hydroxyl groups. Figure 3b further elucidates these molecular interactions. The small peak around ~3480 cm^−1^ for MS corresponds to O–H bending, which becomes more pronounced and stronger after modification with the PDA nanocoating, indicating the formation of additional hydrogen bonds. As shown in Figure 3b, the increasing intensity at approximately 1600 cm^−1^ indicates the formation of the PDA nanocoating. This peak is significant and confirms the presence of PDA on the surface. Figure 3c shows the crystalline structure of both the MS and MS@PDA nanocomposites. The MS exhibits prominent diffraction peaks at approximately 21°, 27°, and 28°, indicating that the confined crystal and mica elements predominantly present in the MS have a typical laminated structure [15,18]. Following surface modifications and acid/alkali treatments, the diffraction peaks remained largely unchanged, which attests to the high chemical structural stability and corrosion resistance of the MS-based composites, making them suitable for a long-term sustainable disinfection system.

### 3.3. Interfacial Bioadsorption Performance of MS@PDA

**Figure 4:**
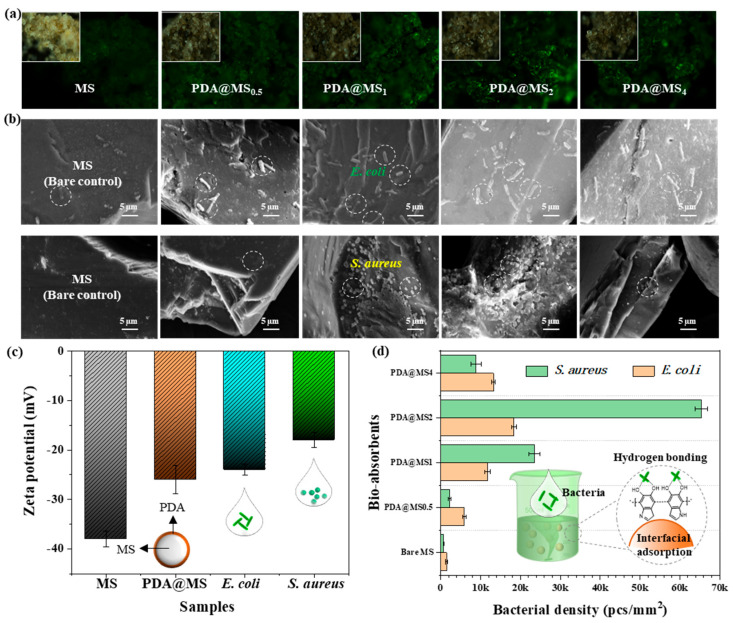
(**a**) Optical images of different MS@PDA samples after adsorption tests using fluorescent-dyed bacteria, captured under bright and UV light conditions, respectively. (**b**) SEM images of various MS@PDA samples after a 30 min adsorption test with two bacterial types (*E. coli* and *S. aureus*, at a concentration of 10^8^ CFU/mL; The “*” represents the repeating structural units of the polymer molecule; the circles indicate the bacteria adhered to the material surface). (**c**) Zeta potential measurements. (**d**) Comparative analysis of bio-adsorption efficiencies for the two model bacterial strains based on the density of bacteria adhered to the surfaces.

To verify the enhanced bio-adsorption performance of the PDA nanocoating, a static adsorption experiment was performed using *E. coli* and *S. aureus*, two common microorganisms found in wastewater. The bacteria were co-incubated for 30 min at room temperature. For visualization, both bacterial strains were stained using an acridine orange/ethidium bromide fluorescent nuclear staining method before the adsorption process [27]. Figure 4a shows the fluorescence images of surfaces coated with *E. coli* following the static adsorption process. All MS@PDA samples exhibited noticeably higher fluorescent intensities compared to bare MS, indicating that the PDA nanocoating facilitated greater bacterial adhesion to the particle surfaces. Notably, fluorescence intensities increased progressively with longer reaction times, reaching a peak for MS@PDA_2_. The topographies after adsorption with bacteria were captured using SEM measurements.

Figure 4b shows that the bare control of MS demonstrated minimal adsorption capability for the bacteria, with no substantial bacterial presence detected on the particle surface. In contrast, after PDA modification, a substantial number of bacteria appeared on the surface morphologies (indicated by white circles) for both *E. coli* and *S. aureus,* indicating that the PDA nanocoating possesses a strong adsorption capacity for these two bacterial types. Furthermore, the count of bacteria adhered to the particle surfaces exhibited a similar trend, initially increasing and then decreasing. These findings are aligned with the variations observed in the fluorescent optical signals, which could be explained by the PDA nanocoating enhancing the bio-adsorption properties and the long processing time, which was assumed to lead to the spontaneous degradation of the surface-adhered bacteria based on the typical compositions of MS (e.g., metal oxides) and catechol groups endowed with antibacterial properties (e.g., ROS) [28]. The surface zeta potential of MS, MS@PDA, and the two bacterial strains was measured in a PBS buffer solution (pH ~7.0, 0.1 M), with results depicted in Figure 4c. After the PDA nanocoating was applied, the zeta potential of MS decreased from 38 to 26 mV, indicating that the reduced surface charges would result in weaker repulsion towards the negatively charged bacteria. Furthermore, the abundant catechol groups in the PDA nanocoating provided excellent film-forming properties and strong adhesion capabilities, either to the MS or the bacterial surfaces, owing to robust hydrogen bonding. This characteristic considerably contributed to bacterial adsorption. Figure 4d depicts the calculations for bacterial density and corresponding adsorption efficiency. The inset images schematically illustrate the PDA nanocoating-assisted adsorption of bacteria and the potential interfacial bio-adsorption mechanism. It was confirmed that the PDA nanocoating considerably enhanced the interfacial bio-adsorption performance of MS, achieving a bacterial density of 6.6 k pcs mm^−2^ for *S. aureus* and 1.8 k pcs mm^−2^ for *E. coli*. These findings suggest that MS@PDA_2_ has considerable potential as a versatile adsorbent for wastewater disinfection.

**Figure 5:**
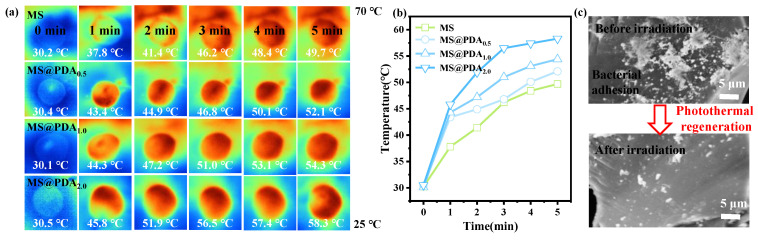
(**a**) The photothermal activities of the nanohybrids were assessed using 808 nm near-infrared light (NIR, 1.0 W) and irradiated for 5 min. (**b**) Surface temperatures were recorded as a function of time. (**c**) Surface microstructural observations were performed before and after exposure to IR light irradiation.

This section evaluates the photothermal efficiencies and regeneration capabilities of the MS@PDA nanocomposites. Thermal images were captured at each time point using a digital thermometer (Figure 5a), with the resulting time-dependent surface temperatures summarized in Figure 5b. The PDA-coated MS exhibited a rapid increase in temperature compared to the bare MS sample after 5 min of NIR exposure, and the photothermal efficiencies could be further adjusted by varying the thickness of the PDA nanocoating. These observations can be attributed to the synergistic effects of surface plasmon resonance stemming from the metallic oxide in the MS and the PDA nanocoating. The temperature of MS@PDA_2_ increased to 58.3° over 5 min, indicating its potential application in the photothermal regeneration of absorbents through a temperature-induced bacterial degradation mechanism (Figure 5c). Overall, this section demonstrates that MS@PDA can serve as a new high-performance bio-adsorbent and antibacterial material, with PDA acting as an intermediate coating to bind bacteria to MS substrates, followed by in situ bacterial elimination, owing to its inherent antimicrobial properties and enhanced photothermal effects.

**Figure 6:**
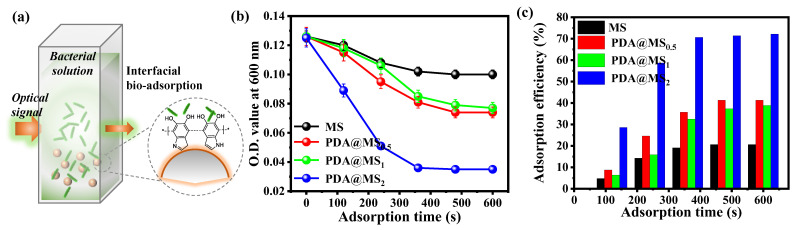
(**a**) Schematic representation of bacterial adsorption performance measured using the static adsorption method. (**b**) Optical density (O.D.) values and (**c**) removal percentage as a function of adsorption times over 10 min. The “*” represents the repeating structural units of the polymer molecule.

In this section, *S. aureus* was selected as a model bacterial pollutant to assess the adsorption activity of MS@PDA. A static adsorption experiment was performed by adding 200 µL of *S. aureus* with a concentration of 10^8^ CFU/mL into a 5 mL buffer solution and magnetic stirring with 0.25 g of MS@PDA at 100 rpm for 10 min. The O.D. of the resulting solution at a wavelength of 600 nm was recorded at each specific time point using UV–vis to determine the bacterial concentration and removal efficiency (Figure 6a). An O.D. value of 1 is defined as equivalent to 1 × 10^9^ CFU/mL. As illustrated in Figure 6b, the O.D. value exhibited a downward trend in the first 6 min before stabilizing, indicating that the bacteria quickly adhered to the surfaces of the MS@PDA particles, gradually reaching a saturated state. However, it should be noted that the mineral stones themselves may exhibit dissolution behavior, which could contribute to their antibacterial effects and result in a decrease in the O.D. value. Figure 6c presents a comparison of the adsorption rates between the MS and MS@PDA samples over a period of 10 min, revealing that the adsorption rates peaked at the initial stage, highlighting the rapid adhesion of bacteria to the particle surfaces. The MS@PDA_2_ demonstrated an adsorption efficiency of 28.5% over 100 s, which is 6.0 times greater than that of the bare MS samples (4.76%). Based on the reduction in the O.D. value and the decolorization of the bacterial solution, both the bare MS and MS@PDA_2_ reached a maximum removal efficiency of 21% and 72%, respectively, after more than 10 min of incubation. All PDA-doped MS samples exhibited relatively higher bacterial adsorption efficiencies compared to the bare sample. These findings suggest that the benefits of PDA nanocoating include considerable energy savings, which are key factors in assessing the viability of stone-based adsorbents for treating bacteria-infected wastewater. Finally, to assess the number of bacteria remaining on the media after adsorption, optical and SEM images were captured to illustrate the regrowth of two bacterial strains on the MS@PDA surfaces after 10 min of exposure to the bacterial solutions. The fluorescence images and surface observations reveal that the bacterial densities did not considerably differ from those of the pristine samples. Additionally, after coculturing for 48 h, only a minimal number of bacteria were detected on the material surfaces, further confirming the inherent antibacterial properties of the MS@PDA, with some debris still present. These results are in good agreement with the previous reports that the mineral component of MS was able to confer inherent antimicrobial activities.

### 3.4. Synergistic Effect of Bio-Adsorption and Cycling Infiltration on Bacteria-Infected Water Treatment

**Figure 7:**
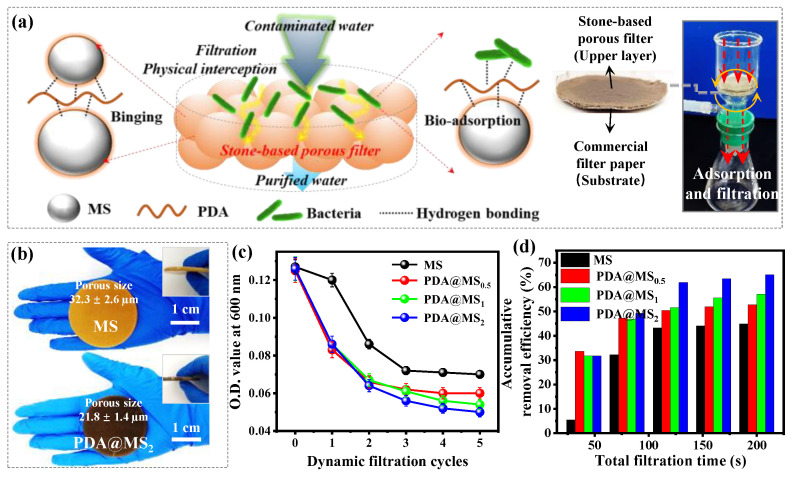
(**a**) Schematic representation of a conventional dead-end filtration device operating under pressure (0.1 MPa) and the potential dynamic filtration adsorption pathway for treating bacteria-infected water. (**b**) Optical images of the MS and MS@PDA functioning as porous filter devices (insets show the averaged porous films of free-standing samples). (**c**) The optical densities and (**d**) bacterial adsorption rates in relation to the dynamic filtration cycles/times (approximately 40 s per cycle).

This section assesses the synergistic disinfection performance achieved by combining interfacial bio-adsorption with dynamic filtration processes using the stone-based porous filter membrane (Figure 7a). *S*. *aureus* was specifically chosen as the indicator microorganism, and a bacterial solution with a concentration of 10^8^ CFU/mL was used as the feed water to simulate typical bacterial levels found in water purification systems. It is important to mention that the bare MS alone was unable to be formed into a stable film and lacked long-range mechanical strength. Consequently, the incorporation of the PDA nanocoating was shown to considerably enhance the binding interactions among the particles, leading to a relatively well-packaged porous film with commendable mechanical strength (Figure 7b). The pore size distributions of the stone-based porous filter membranes were evaluated using a capillary flow porometer, yielding average pore sizes of 32.3 ± 2.6 µm, 28.5 ± 1.7 µm, 25.1 ± 2.9 µm, and 21.8 ± 1.4 µm for the MS, MS@PDA_0.5_, MS@PDA_1_, and MS@PDA_2_, respectively. Notably, the mesoporous structure in the MS could not be detected and was beyond the capabilities of the PMI measurements. These findings indicate that the porous structure can be influenced by the particle sizes of the MS raw materials and can be further modified through the formulation of the PDA nanocoating. Upon flowing through the stone-based filter membrane (~40 s per cycle), as illustrated in Figure 7c, a fast decline in the O.D. value was observed in both bacterial solutions, implying that certain bacterial strains were effectively removed through dynamic cyclic filtration and interfacial adsorption processes. To evaluate the long-term performance and large-scale treatment capacity of this new porous filter, a cyclic flow-through dynamic adsorption filtration and disinfection apparatus has been implemented [29]. Figure 7d shows that the removal efficiencies of the PDA-modified porous membranes reached up to 30% during the first round of filtration over 40 s, which is 5.2 times greater than that of the bare MS samples (5.8%). Comparing the filtration trials with the static adsorption experiments suggests that physical interception plays a dominant role in the initial stage, based on the principle of size-dependent separation. Typically, larger bacterial strains are more easily trapped in the stone-based porous network, leading to smaller or blocked pores; consequently, the rejection capacity is expected to increase, accompanied by a decrease in water flux. The filtration time for each cycle remained consistent at approximately 40 s, and the water flux showed no substantial changes, indicating that the porous structure was not completely blocked. The cumulative removal efficiencies increased progressively over five cycles, demonstrating a synergistic effect between cyclic filtration and long-term adsorption processes.

**Figure 8:**
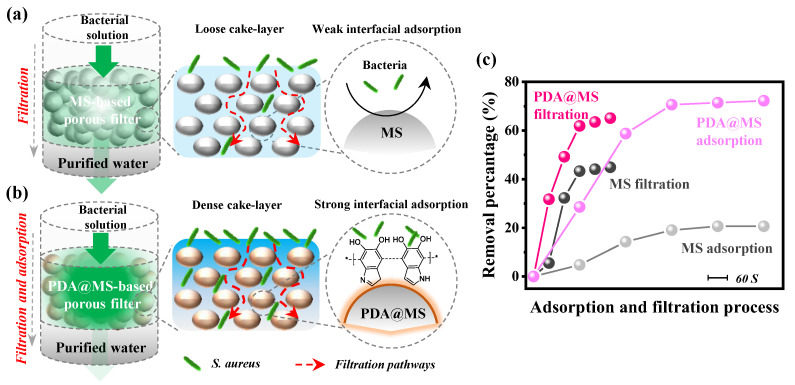
Schematic illustrating the potential dynamic filtration and interfacial adsorption mechanisms of (**a**) MS and (**b**) MS@PDA-based porous filters for bacterial purification of water. (**c**) Comparison of bacterial removal efficiencies between static adsorption and dynamic filtration tests. The “*” represents the repeating structural units of the polymer molecule.

The proposed mechanisms of the bare MS and MS@PDA-based porous filter membranes for dynamic filtration and adsorption in sustainable wastewater disinfection are shown in Figure 8a,b. As expected, the bacterial concentration decreased rapidly during the initial phase, subsequently stabilizing as bacteria accumulated to a certain level through cyclic filtration. The incorporation of the PDA nanocoating not only provides exceptional bio-adsorption performance for bacteria (e.g., *S. aureus*) but also improves binding interactions with adjacent particles, resulting in a mechanically stable stone-based porous filter membrane suitable for sustainable wastewater disinfection. The bacterial removal efficiencies from the static adsorption and dynamic filtration tests (5 cycles) are summarized in Figure 8c. The static adsorption method demonstrates considerable removal efficiency; however, it is time-consuming and has a low throughput. In contrast, the filtration tests revealed a rapid increase in removal efficiency in the first 60 s, achieving final bacterial capture rates of 68% for MS@PDA_2_ and 43% for bare MS. This enhanced bacterial removal can be attributed to the bacteria-enriched filtration process and PDA-assisted interfacial bio-adsorption. Consequently, this innovative type of natural stone-based material represents a sustainable purification medium with high efficiency for water disinfection treatment.

## 4. Conclusions

A new bio-adsorbent and porous filter device designed for high-efficiency wastewater disinfection was developed from a naturally abundant MS, enhanced through surface modification with an active PDA nanocoating. Experimental results show that the PDA nanocoating can be simply prepared via self-polymerization of DA monomers on the MS surfaces, resulting in considerably improved adhesion properties and bio-adsorption performance against two types of bacteria, with surface bacterial densities of 6.6 k pcs mm^−2^ for *S. aureus* and 1.8 k pcs mm^−2^ for *E. coli*. To illustrate the high throughput and efficiency in the continuous removal of bacteria from wastewater, the filtration performance of the MS@PDA-based porous filter device, featuring an average pore size of 21.8 ± 1.4 µm and porosity of ~83, was evaluated through a cycling filtration experiment. The cumulative removal efficiencies observed during cycling filtration were 42% and 68% for bare MS and MS@PDA, respectively. The underlying mechanisms were identified as a result of the synergistic effects of interfacial bio-adsorption and filtration. Furthermore, the bacteria captured on the surface could be killed using the enhanced photothermal effects of the PDA nanocoating and the inherent antimicrobial properties of the mineral stone. Thus, this study provides a green and universal approach to constructing an MS-based advanced bio-adsorbent and filter for sustainable water purification.

## Data Availability

The original contributions presented in the study are included in the article, and further inquiries can be directed to the corresponding author.
